# Insects Associated with Declining Riparian Black Alder (*Alnus glutinosa*) Stands: Assemblage Structure, Within-Season Patterns, and Distance–Zone Patterns in the Utrata and Łutownia River Valleys

**DOI:** 10.3390/insects17060551

**Published:** 2026-05-27

**Authors:** Konrad Wilamowski, Tomasz Pawłowicz, Monika Puchlik, Tomasz Oszako

**Affiliations:** 1 Institute of Forest Sciences, Faculty of Civil Engineering and Environmental Sciences, Białystok University of Technology, ul. Wiejska 45E, 15-351 Białystok, Poland; m.puchlik@pb.edu.pl; 2 Forest Protection Department, Forest Research Institute, ul. Braci Leśnej 3, 05-090 Raszyn, Poland; t.oszako@ibles.waw.pl

**Keywords:** riparian forests, *Alnus glutinosa*, alder decline, insect assemblages, saproxylic insects, dead wood, riparian habitat mosaic

## Abstract

Insect assemblages associated with declining riparian black alder (*Alnus glutinosa*) stands were compared at the community level between two lowland river valleys in Poland that differ in anthropogenic transformation: the near-natural Łutownia River valley within the Białowieża Forest and the urbanised Utrata River valley in the Warsaw metropolitan area. Insects were sampled using active hand sampling and Barber pitfall trapping along a short-distance gradient from the channel during repeated monthly field campaigns in summer and early autumn. The near-natural valley supported a richer assemblage, with a stronger representation of forest-associated, saproxylic and aquatic taxa, whereas assemblages in Utrata contained a higher proportion of widespread disturbance-tolerant taxa. In both valleys, diversity was highest at an intermediate distance from the channel margin, and the between-valley contrast was more pronounced than the within-season shifts visible in the campaign summaries. The results identify a clear assemblage contrast between the two valleys, a consistent intermediate-distance diversity maximum, and only modest within-season variation in pooled campaign totals. These findings indicate that structurally complex riparian alder forests with retained dead wood are associated with richer and more taxonomically varied insect assemblages; microhabitat-related interpretations are retained as cautious sampling-point-scale associations because tree-level attributes and individual microhabitat categories were not quantified separately.

## Simple Summary

Riverside black alder forests are being altered by stand decline, while many river corridors are increasingly affected by urban development and roads. We compared insects living in declining black alder (*Alnus glutinosa*) forests along two lowland rivers in Poland: the near-natural Łutownia River in the Białowieża Forest and the urbanised Utrata River near Warsaw. From June to September 2024, insects were sampled by hand and with pitfall traps at the channel margin and at 15 and 30 m from the river. The near-natural valley had a greater variety of recorded insects, including more species and groups linked to dead wood and wet or aquatic habitats. In the urban valley, the insect fauna was mainly composed of common species that can live in disturbed places. Overall, the results suggest that alder forests with a varied structure and retained dead wood provide better conditions for a richer insect fauna, including insects connected with decaying wood and water. Differences observed between sampling points are interpreted as local patterns within the riverside forest environment, rather than as separate measured effects of each small habitat feature.

## 1. Introduction

Declining riparian stands dominated by black alder (*Alnus glutinosa*) increase structural complexity by adding dead wood and creating associated microhabitats. These features influence microclimatic conditions, flow dynamics and sediment retention, and they support diverse invertebrate groups [1,2]. Large fragments of dead wood can also provide habitat for aquatic insect larvae by offering shelter from predators and feeding substrates [3,4].

Black alder is widespread in Central European riparian habitats and plays an important role in nutrient cycling, especially nitrogen, through symbiosis with bacteria of the genus *Frankia*, which fix atmospheric nitrogen [5,6]. In addition, alder leaf litter constitutes a valuable detrital resource that supports trophic pathways in running-water ecosystems by promoting the development of detritivorous taxa [7]. Previous studies have shown that the structural and nutritional heterogeneity of alder-dominated riparian stands can promote distinctive insect assemblages, including both terrestrial (e.g., Carabidae, Staphylinidae) and aquatic indicator taxa (e.g., Tipulidae, Chironomidae, Simuliidae) [8,9]. However, available knowledge remains fragmentary. Studies of declining alder stands have focused mainly on selected saproxylic beetles [10], whereas many riparian insect studies have examined aquatic assemblages, biomonitoring, or land-use effects at the stream level rather than whole insect assemblages associated with declining riparian black alder stands [11,12,13]. Consequently, it remains insufficiently understood how assemblages linked to the riparian habitat mosaic around declining alder stands vary between valleys and along short distances from the channel.

Riparian zones also provide important regulatory functions. Forested riparian strips can reduce pollutant runoff, limit nutrient loading to surface waters, stabilise water temperature and mitigate bank erosion [11,14]. The effectiveness of these functions depends on buffer width and structure, and even relatively narrow forested strips (approximately 10–30 m) may measurably improve water quality and increase the occurrence of indicator species [15,16]. Water quality remains a major determinant of aquatic insect community structure, with physicochemical parameters such as dissolved oxygen, nitrate and phosphate concentrations, pH, conductivity and temperature influencing the persistence of sensitive taxa [12,17]. Mayflies (Ephemeroptera), stoneflies (Plecoptera) and caddisflies (Trichoptera), collectively referred to as EPT taxa, are among the most commonly used bioindicators in ecological assessments of European streams and rivers [18]. Such studies are fundamental for assessing ecological status, but they usually treat aquatic indicator groups or physicochemical conditions as the primary response [12,17,18]. They therefore do not show how insect assemblages associated with declining riparian alder stands vary when terrestrial, saproxylic and aquatic taxa are considered together along a short gradient from the river channel and between valleys that differ in anthropogenic transformation.

In Poland, the Łutownia and Utrata rivers provide a useful contrast between riparian valleys that differ strongly in anthropogenic transformation. The Łutownia, within the Białowieża Forest, flows through largely natural forest habitats with substantial dead wood resources and comparatively stable hydrological conditions. The studied reach is only weakly transformed, retains a relatively continuous riparian alder belt and contains frequent standing and fallen dead wood; such settings often support diverse saproxylic and aquatic insects, including rare and relict species [10,19]. By contrast, the Utrata valley on the outskirts of Warsaw is strongly affected by anthropogenic pressure. Channel regulation and surrounding urban land use have produced a more fragmented riparian corridor, and dead wood is present only locally and in smaller amounts than in the Łutownia valley. Nevertheless, remnant fragments of riparian alder forest and local accumulations of dead wood may still support insect assemblages and partially buffer urbanisation impacts [13,20].

Against this background, the present study compares insect assemblages associated with declining riparian black alder (*Alnus glutinosa*) stands in two lowland river valleys in Poland that differ markedly in anthropogenic transformation: the near-natural Łutownia River valley within the Białowieża Forest and the urbanised Utrata River valley in the Warsaw metropolitan area. Unlike most previous studies, which have focused either on selected saproxylic beetles [10] or on aquatic indicators and physicochemical conditions [12,13], our design combines a between-valley comparison with sampling along a short-distance gradient from the channel over the June–September study period. Insects from the vegetation layer, dead wood and ground layer were analysed jointly as components of the riparian habitat mosaic associated with declining alder stands rather than as separately quantified microhabitat categories; accordingly, the study addresses assemblage patterns at the sampling-point scale and does not test the effects of individual tree attributes or specific microhabitat variables.

Accordingly, the study had three objectives: to compare insect assemblages associated with declining riparian alder stands between the near-natural and urbanised valleys; to examine how richness, abundance and assemblage composition varied along the short-distance gradient from the channel margin; and to describe within-season variation over the sampling period. We expected the near-natural Łutownia valley to support a richer assemblage with a greater contribution of forest-associated, saproxylic and aquatic taxa than the urbanised Utrata valley, and we anticipated spatial differentiation among distance zones. Because insects were analysed at the sampling-point scale within a composite riparian habitat mosaic, microhabitat-related interpretations are framed as assemblage associations with that mosaic.

## 2. Materials and Methods

The study was conducted in two lowland river valleys in Poland that represent contrasting environmental settings and markedly different degrees of anthropogenic transformation: the Łutownia River in the Białowieża Forest (northeastern Poland) and the Utrata River in the Warsaw metropolitan area (central Poland).

The Łutownia River is a left-bank tributary of the Narewka River and flows through the Białowieża Forest, one of the most valuable forest complexes in Europe and a UNESCO World Heritage property. The study reach is situated in northeastern Poland, within the Bielsk Plain mesoregion, and the river has retained a largely natural planform with numerous meanders and a well-developed riparian zone.

Riparian forests along the Łutownia are dominated by black alder (*Alnus glutinosa*) and form moisture-rich habitats that, during field surveys, contained frequent standing and downed dead wood and a diverse vertical structure. The surveyed Łutownia reach represented a near-natural and only weakly transformed riparian system: standing dead alders and fallen trunks were frequent within the sampled reach, the shrub and herb layers were well developed, and the riparian forest formed a relatively continuous belt along the channel. Natural disturbance and succession processes were pronounced, including alder dieback, fallen trunks and locally well-developed organic horizons, creating a mosaic of saproxylic substrates and favourable conditions for diverse riparian insect assemblages, including forest-associated and aquatic taxa [10,19]. These field observations define the environmental context of the sampled reach and were used to support the interpretation of assemblage patterns at the sampling-point scale.

The Utrata River is a right-bank tributary of the Bzura River in the Mazovia Province. The study area comprised sections of the middle course of the river near the boundary between Pruszków and Piastów. The valley lies in the Łowicko-Błońska Plain mesoregion (Central Mazovian Lowland macroregion) within the Warsaw agglomeration and is characterised by extensive spatial transformation associated with urbanisation and anthropogenic pressure [20].

The Utrata channel has been regulated in many places and is surrounded by arable land, urban development and impacted catchments. During field surveys, the studied Utrata reach represented a markedly transformed suburban riparian system: the alder strip was narrower and more fragmented, direct signs of channel modification were evident, and dead wood occurred mainly as local, discontinuous accumulations rather than as a frequent, spatially continuous feature. Nevertheless, fragments of riparian alder forest with dying trees persisted locally, and structural features characteristic of *Alnetum glutinosae* habitats remained observable. These remnants formed the local alder habitat context sampled in the Utrata valley; structural observations were used as site context for interpreting assemblage patterns at the sampling-point scale.

### 2.1. Location and Spatial Distribution of Sampling Points

To assess spatial variation in insect assemblage composition, nine sampling points were established: three along the Utrata River and six along the Łutownia River. Placement reflected valley characteristics and the study objectives. The coordinates listed in Table 1 denote approximate reference locations for the sampled areas and are not intended to identify the exact position of individual traps or hand-sampling locations.

**Figure 1:**
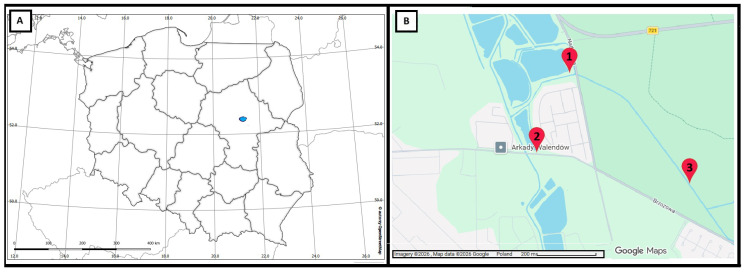
Approximate locations of the sampling points on the Utrata River within the Warsaw metropolitan area. In panel (**A**), the blue circle indicates the approximate location of the study area within Poland. The numbers shown in the figure denote the sampling points. Panel (**A**) is based on OpenStreetMap data from OpenStreetMap contributors, made available under the Open Database License (ODbL) [21]. Panel (**B**) is based on Google Maps content and is used in accordance with Google Geo Guidelines [22]; the required attribution to Google and any indicated third-party data providers is retained directly on the map image.

In the Utrata valley (Figure 1), sampling points were positioned within an urbanised suburban landscape to capture variation in surrounding land use and channel modification. Two sampling points were located in strongly transformed reaches near Pruszków and Piastów, whereas one sampling point was located in a remnant riparian alder fragment with comparatively developed riparian vegetation. At the reach scale, however, the Utrata sites remained embedded in a regulated and fragmented riparian corridor, and the occurrence of dead wood was local rather than continuous. This arrangement supports comparisons within the Utrata River and with the near-natural Łutownia reference valley (Table 1).

**Figure 2:**
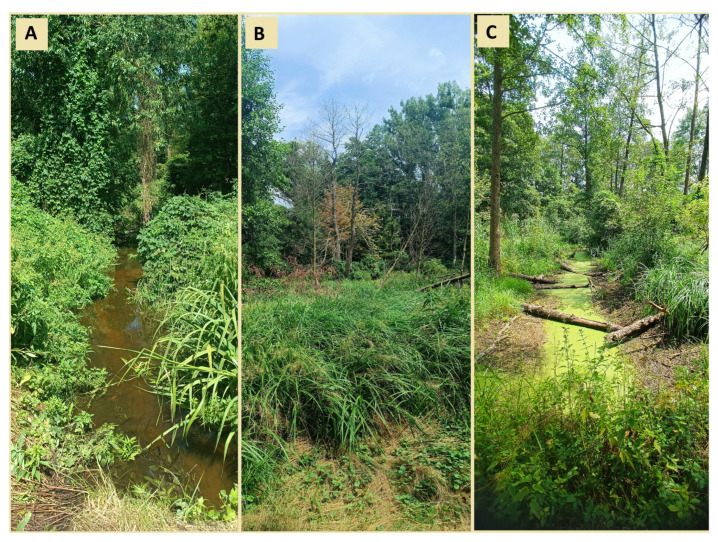
Photographs of the three sampling points (PU1–PU3) in the Utrata River valley, illustrating the immediate habitat context around the sampling locations within a transformed suburban riparian corridor. Panel (**A**) shows sampling point PU1; panel (**B**) shows sampling point PU2; and panel (**C**) shows sampling point PU3. Local thickets or individual fallen trees visible in the photographs reflect point-scale conditions and are not intended as a quantitative representation of valley-scale naturalness or dead wood abundance. Photographs: K. Wilamowski.

The Utrata River valley lies in the suburban zone of the Warsaw agglomeration and is subject to substantial anthropogenic pressure. Three sampling points (PU1–PU3) were identified in the field to represent variation in disturbance intensity and surrounding land use. Photographs illustrate the local habitat context at each sampling point (Figure 2).

**Figure 3:**
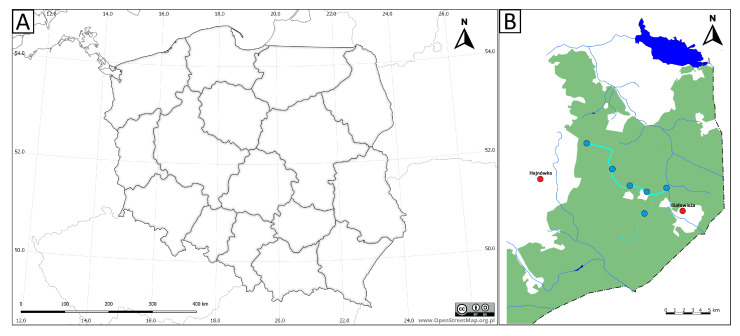
Approximate locations of the sampling points along the Łutownia River within the Białowieża Forest, from upstream sections to downstream reaches near the confluence with the Narewka River. Panel (**A**) shows the general location of the study area within northeastern Poland; the blue circle indicates the approximate location of the Łutownia River study area. Panel (**B**) shows the detailed distribution of the sampling points along the Łutownia River within the Białowieża Forest.

In the Łutownia valley (Figure 3), sampling points were distributed along the river course from upstream sections to downstream reaches near the confluence with the Narewka River. Located within the Białowieża Forest, this layout supports a descriptive assessment of variation in insect assemblages across a less transformed and more continuous forested valley, with structurally complex riparian alder stands and frequent dead wood [10,19].

**Figure 4:**
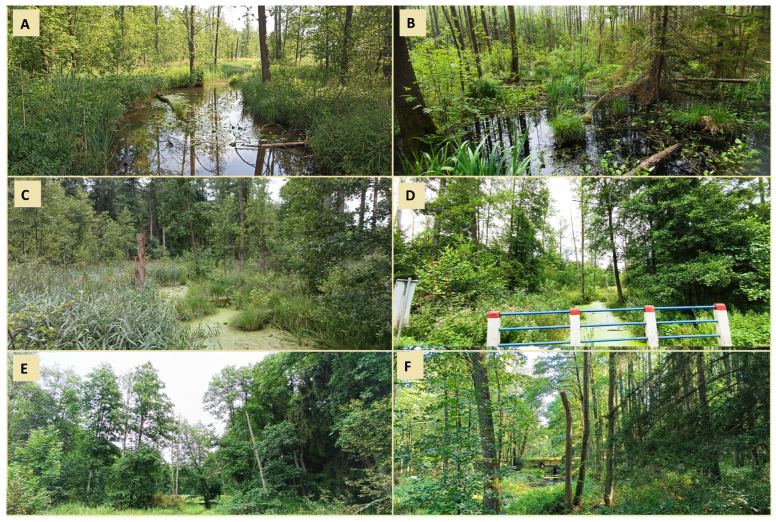
Photographic documentation of sampling points PŁ1–PŁ6 in the Łutownia River valley, illustrating characteristic features of riparian alder habitats. PŁ1 (**A**), PŁ2 (**B**), PŁ3 (**C**), PŁ4 (**D**), PŁ5 (**E**), and PŁ6 (**F**). Approximate reference locations and rounded WGS 84 coordinates for these sampling points are provided in Table 1. Photographs: K. Wilamowski.

The photographs document point-level variation within the Łutownia riparian alder corridor, including the local structure of forested banks, understorey vegetation and declining-alder habitats sampled at PŁ1–PŁ6 (Figure 4).

Sampling points were selected in riparian stands where black alder (*Alnus glutinosa*) showed visible field signs of decline or recent mortality, including crown thinning or dieback, dead branches, bark loss or loosening, and the presence of standing or fallen dead alder stems. These criteria were applied qualitatively during site selection and define the stands referred to throughout the manuscript as declining alder stands. The design characterises insect assemblages associated with declining alder stands and surrounding riparian substrates at the sampling-point scale; quantitative tree-condition scoring, decay-stage classification, pathogen diagnostics and standardised tree-size measurements were outside the scope of the field protocol.

### 2.2. Entomological Material Collection

Two complementary sampling approaches were used: active hand sampling and Barber pitfall trapping. At each visited sampling point, both methods were applied within three distance zones measured from the channel margin: 0 m, 15 m and 30 m. The operational sampling unit was therefore the sampling point × distance–zone combination, which supports comparisons of assemblage structure among river–distance strata. Window traps, bark traps and emergence traps were not used, so the sampled assemblage represents insects detectable by hand searching and pitfall trapping within the accessible riparian zone.

For field sampling, microhabitats were defined broadly as accessible substrates and microsites within the riparian area around each sampling point, including herb and shrub vegetation, the soil and litter surface, dead-wood surfaces, loose bark and accessible under-bark spaces, and fungal fruiting bodies or other dead-wood-associated structures when present. These elements were sampled collectively as the riparian habitat mosaic associated with declining alder stands. Because sampling effort was not partitioned into fixed quotas for individual microsite types, microhabitat-related interpretations in the manuscript refer to assemblage associations within this composite habitat mosaic rather than to separately modelled effects of individual microhabitat categories.

Active hand sampling used an entomological net and forceps to collect insects from accessible vegetation, dead wood, exposed bark surfaces, and the soil surface during daytime field visits. No fixed sampling height was imposed, and material was collected only from microsites that could be reached from ground level, so upper-trunk and canopy habitats were not sampled. Individuals that could be identified reliably in the field or from photographs were recorded, photographed where useful, and released. Non-protected specimens that required later verification were retained individually in labelled microcentrifuge tubes or vials and identified using morphological characters and standard keys; selected difficult material was additionally checked by taxonomic specialists. Records are reported at the species level where secure identification was possible and otherwise at the lowest reliable taxonomic rank, and no artificial morphospecies codes were used in the final dataset. Butterflies were recorded from photographs only, and three observations that could not be identified securely were excluded from the final checklist and analyses. Legally protected species were documented photographically and released, whereas retained non-protected material is currently held in the private reference collection of Konrad Wilamowski, housed at the Institute of Forest Sciences, Białystok University of Technology.

Barber-type pitfall traps were installed to sample surface-active ground-dwelling fauna, with one trap placed in each distance zone at every visited sampling point (three traps per visited point during a campaign). The traps consisted of round plastic containers (20 cm diameter, 10 cm deep) covered by simple canopies; the containers were buried flush with the soil surface and filled with saturated NaCl solution plus a small amount of detergent. During each scheduled field campaign, traps at the visited points were emptied, and the collected material was transferred to 70% ethanol and labelled with sampling point, date and distance zone.

### 2.3. Temporal Scope of the Study

Field sampling was conducted once per month from June to September 2024. All three Utrata sampling points (PU1–PU3) were surveyed during each campaign, so each Utrata point was visited four times during the season. In the Łutownia valley, three sampling points were surveyed per campaign in two alternating subsets (PŁ1–PŁ3 in June and August; PŁ4–PŁ6 in July and September) to accommodate field logistics and access constraints; thus, each Łutownia point was visited twice. Consequently, the total number of point visits across the season was the same in both rivers (12 per river), although the number of unique sampling points differed (three in Utrata and six in Łutownia). For this reason, month-to-month comparisons in Łutownia partly combine temporal change with differences among sampling points and are treated as descriptive only.

During each campaign, both collection methods were applied in each distance zone (0, 15 and 30 m) at every visited sampling point. Active hand sampling consisted of one daytime search of the accessible riparian area in each distance zone during that visit, and the corresponding pitfall traps were checked and emptied during the same campaign. Sampling dates were standardised as far as practicable to comparable weather conditions, with no precipitation or strong wind and temperatures conducive to insect activity. In the Utrata valley, fieldwork was usually completed between 10:00 and 13:00 on a single day. In the Łutownia valley, the three points scheduled for a given campaign were surveyed in the same daytime window, but fieldwork could extend over two consecutive days because of travel time and difficult wet-terrain access. Nominal sampling effort was therefore balanced between rivers in terms of total seasonal point visits, distance zones and methods, although the exact duration of active searching could vary locally with terrain accessibility.

### 2.4. Data Processing and Statistical Analyses

Analyses were conducted in R (v.4.5.3) using a cleaned taxon-by-stratum table derived from the full field dataset. Records were organised by sampling point (PU1–PU3 in the Utrata valley; PŁ1–PŁ6 in the Łutownia valley), distance zone from the channel margin (0, 15 and 30 m), month and sampling method (active hand sampling or pitfall trapping). Most records were identified to species level, but some material—especially within Diptera and Hymenoptera—was retained at the lowest reliable taxonomic rank when species-level identification was not secure. No artificial morphospecies codes were used in the final matrix, and three photographic butterfly observations that could not be identified securely to species were excluded from both the final checklist and the analytical matrix.

For the community-level analyses, counts were pooled across sampling points and methods within each river × distance stratum and, where required for across-month summaries, across months, yielding a six-stratum taxon-abundance matrix (Utrata 0, 15 and 30 m; Łutownia 0, 15 and 30 m). This matrix was used to characterise assemblage structure and between-stratum contrasts across the two valleys and three distance zones. Tree-level measurements and microhabitat-specific covariates were not included in the matrix, so the analyses describe assemblage associations at the pooled stratum scale. The cleaned source-data matrix, supplementary statistical outputs, and reproducible R code are archived in Mendeley Data [23]. Key derived outputs are also provided in the Supplementary Materials from [23].

Throughout the manuscript, sampling point refers to PU/PŁ codes, distance zone refers to the 0/15/30 m classes, stratum refers to the pooled river–distance analytical units, and recorded taxon refers to the analytical units included in the matrix, regardless of whether identification reached species, genus or family level. Table A2 and Table A3 additionally present month-by-month point-level field summaries pooled across sampling methods for each point–distance combination, allowing the pooled community-level patterns to be compared with the raw local field summaries.

For each stratum, we calculated total abundance (*N*), observed taxon richness ($S_{\mathrm{obs}}$), Shannon entropy ($H^{\prime }=-\sum_{i}p_{i}\ln p_{i}$), the Shannon effective number of taxa ($\exp(H^{\prime })$), Simpson diversity expressed as 1−D where $D=\sum_{i}p_{i}^{2}$, inverse Simpson diversity (1/D), Pielou evenness ($J^{\prime }=H^{\prime }/\ln S_{\mathrm{obs}}$), and Good’s coverage ($C=1-F_{1}/N$, where $F_{1}$ is the number of singleton taxa). 95% percentile bootstrap intervals were estimated for richness and the abundance-based diversity indices by multinomial resampling within strata (5000 resamples with total abundance fixed at the observed *N* and taxon probabilities equal to the observed relative abundances). For observed richness, the percentile interval describes the bootstrap resampling distribution under fixed total abundance and may therefore lie below the original observed value. These intervals are reported to show the scale of variation around the observed stratum values.

Untransformed counts were used for richness, diversity and abundance summaries. As a supplementary standardisation of richness, rarefied richness to the minimum stratum abundance was also calculated and is provided in the Supplementary Materials from [23]. For multivariate visualisation, abundance data were square-root-transformed to reduce the influence of highly dominant taxa while retaining quantitative differences among strata. Bray–Curtis dissimilarities were then calculated from the square-root-transformed abundance data, and these dissimilarities were used for an exploratory two-dimensional non-metric multidimensional scaling (NMDS) ordination of the six strata. The NMDS analysis used two dimensions (k=2) and multiple random starts (trymax =500). PCA was not applied because the dataset consisted of sparse taxon counts with many zero values, for which Bray–Curtis-based rank ordination is more appropriate than Euclidean ordination.

Beta diversity was evaluated on presence–absence data using Sørensen dissimilarity and partitioned into turnover ($\beta_{\mathrm{sim}}$) and nestedness ($\beta_{\mathrm{sne}}$) components, with total dissimilarity expressed as $\beta_{\mathrm{sor}}$. Pairwise values were grouped into three comparison categories (Within Utrata, Within Łutownia, Between rivers). For each category and component, the mean and its 95% percentile bootstrap interval were estimated from 2000 resamples. Because pairwise dissimilarities are not statistically independent, these intervals are interpreted as summaries around the observed category means rather than as formal inferential tests. The full set of pairwise values is provided in the Supplementary Materials from [23].

Sample-size-based rarefaction/extrapolation curves were additionally generated with iNEXT to assess sampling completeness, and the corresponding graphical and tabular outputs are provided in the Supplementary Materials from [23]. The statistical workflow, therefore, combined alpha-diversity estimates, bootstrap intervals, rarefaction/extrapolation, beta-diversity partitioning and exploratory ordination to describe assemblage structure across the six aggregated river–distance strata. Monthly variation was summarised from pooled campaign counts, and interpretation focuses on the direction and consistency of patterns across valleys, distance zones and campaigns rather than on fitted repeated-measures or multivariate hypothesis-testing models.

## 3. Results

Across both river valleys, 136 insect taxa were recorded in the final checklist. More taxa were recorded in the Łutownia valley than in Utrata, and the Łutownia assemblage included a broader representation of forest-associated, aquatic and saproxylic taxa, whereas the Utrata assemblage contained a higher proportion of widespread taxa associated with disturbed environments. The cleaned taxon-by-stratum matrix underlying the community-level summaries is archived in Mendeley Data [23]; key derived outputs are also provided in the Supplementary Materials from [23]. This comparison identifies a clear assemblage contrast between the near-natural and urbanised riparian corridors, while the presence/absence summary in Appendix A documents the taxa contributing to that contrast (Table A1).

The raw point-level field summaries show that pooled counts were higher in the Łutownia valley than in Utrata in every campaign, ranging from 428 to 459 individuals per campaign in Łutownia and from 233 to 267 in Utrata (Table A2 and Table A3). In both valleys, the 15 m distance zone had the highest pooled count in each month, matching the intermediate-distance maximum detected in the community-level diversity analyses. Within-river temporal variation in total counts was modest: Utrata showed a shallow July maximum, whereas Łutownia remained comparatively stable from June to September. Changes among campaigns were more apparent in the identity of dominant taxa than in total abundance, and the alternating monthly subsets in Łutownia mean that month-to-month contrasts in that valley are interpreted at the campaign level.

**Figure A1:**
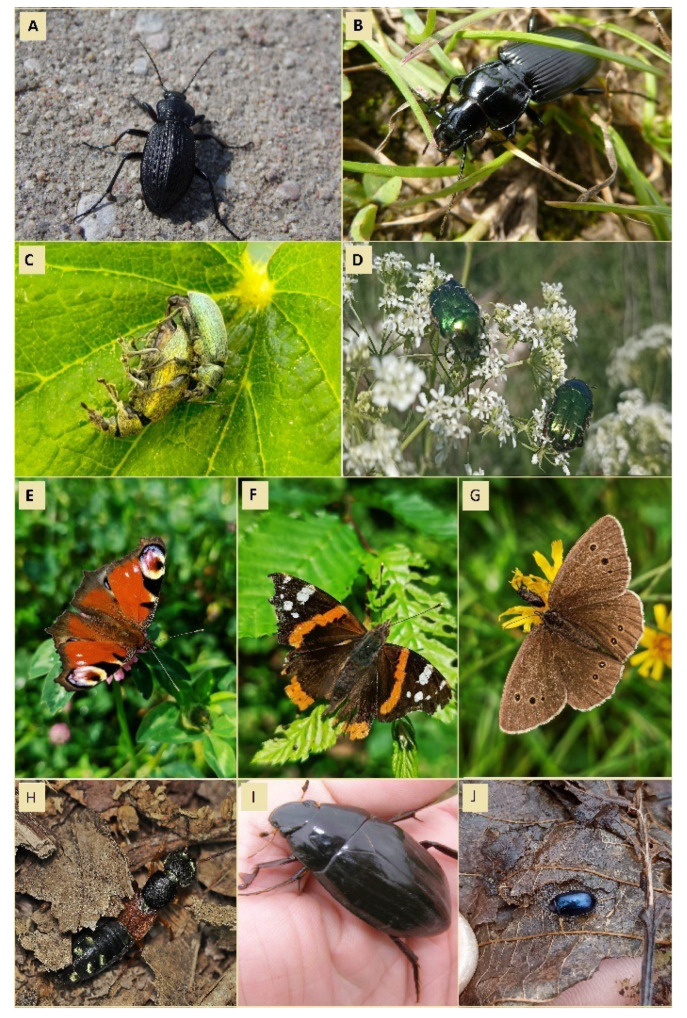
Selected commonly recorded insect taxa from the study areas: *Carabus granulatus* (**A**), *Pterostichus niger* (**B**), *Phyllobius* spp. (**C**), *Cetonia aurata* (**D**), *Aglais io* (**E**), *Vanessa atalanta* (**F**), *Aphantopus hyperantus* (**G**), *Staphylinus erythropterus* (**H**), *Hydrophilus piceus* (**I**), and *Agelastica alni* (**J**). Photographs: K. Wilamowski.

**Figure A2:**
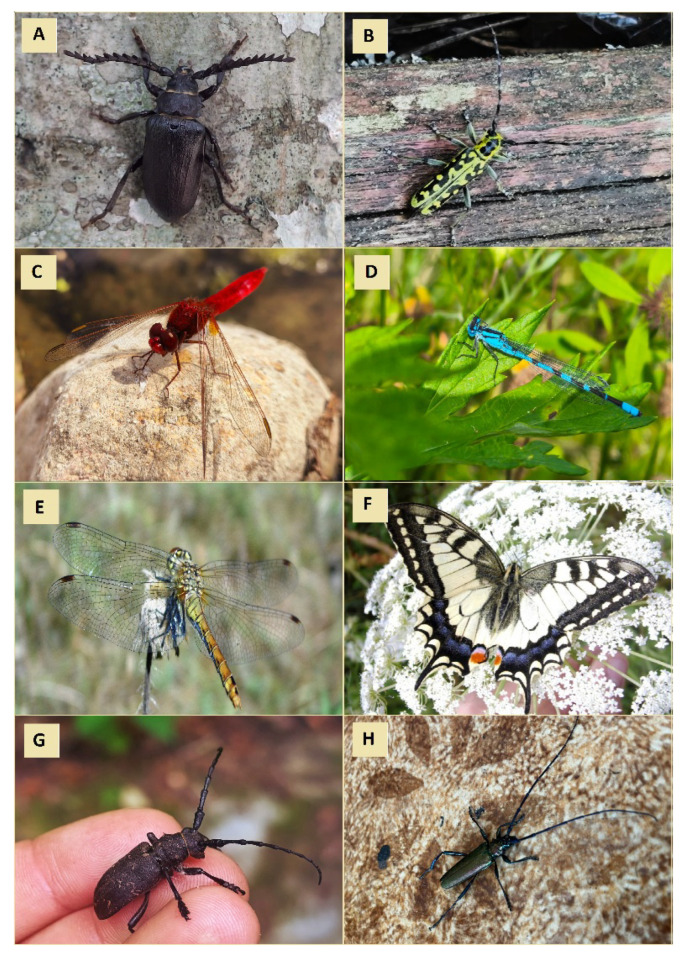
Representatives of less common or characteristic insect taxa observed in the study areas: *Prionus coriarius* (**A**), *Saperda scalaris* (**B**), *Crocothemis erythraea* (**C**), *Enallagma cyathigerum* (**D**), Libellulidae sp. (**E**), *Papilio machaon* (**F**), *Lamia textor* (**G**), and *Aromia moschata* (**H**). Photographs: K. Wilamowski.

The recorded fauna included ground-active beetles, ants, riparian Diptera, Lepidoptera, Odonata, aquatic or semi-aquatic beetles, and taxa associated with dead wood. The photographic plates document this ecological breadth: commonly recorded and characteristic taxa include ground-active, aquatic and herbivorous species, whereas the less frequent records include additional saproxylic, odonate and large-bodied riparian taxa (Figure A1 and Figure A2).

### 3.1. Alpha Diversity

**Figure 5:**
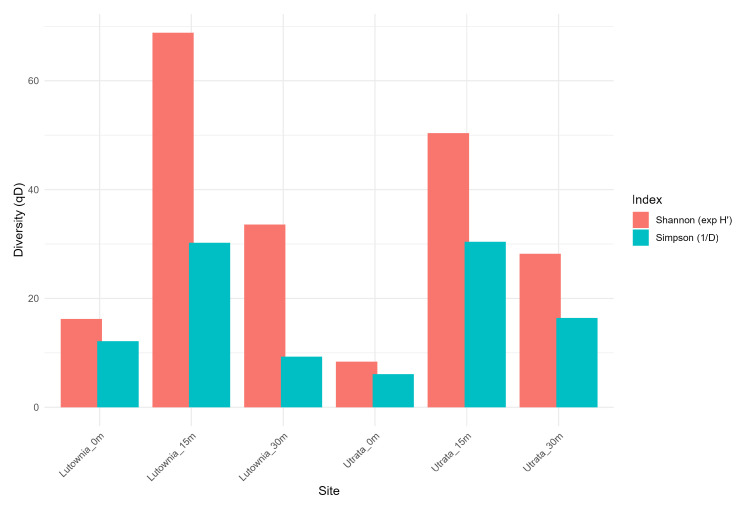
Alpha diversity (Shannon and Simpson). Effective numbers of species ($\exp(H^{\prime })$ and 1/D) by stratum (river × distance zone). Bars enable direct comparison of the two indices across strata.

Alpha diversity differed strongly among river–distance strata, with a consistent intermediate-distance maximum in both valleys (Table 2 and Table 3; Figure 5). Observed richness increased from 12 taxa at 0 m to 75 taxa at 15 m in Utrata and from 25 to 120 taxa in Łutownia; at 30 m, richness remained below the 15 m peak but above the channel-margin stratum in both valleys. Shannon’s effective diversity followed the same pattern, reaching 50.35 in Utrata and 68.80 in Łutownia at 15 m. The 30 m stratum differed between valleys: in Utrata, effective diversity remained intermediate between the 0 and 15 m strata, whereas in Łutownia the lower evenness and inverse Simpson diversity at 30 m indicated stronger dominance by a subset of taxa despite high observed richness. Good’s coverage was high in every stratum (0.985–0.994), indicating that the main diversity contrasts are unlikely to reflect major differences in sampling completeness at the analytical resolution used here. Bootstrap intervals further show that the intermediate-distance maximum was the dominant alpha-diversity pattern across the pooled strata; for observed richness, the percentile interval reflects the bootstrap resampling distribution and does not necessarily bracket the original point estimate (Table 3).

### 3.2. Sampling Completeness and Rarefaction/Extrapolation

**Figure 6:**
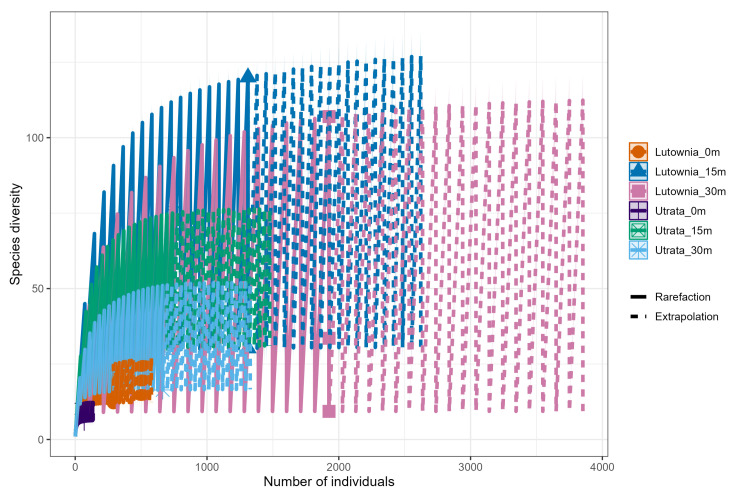
Sample-size-based rarefaction/extrapolation curves for the six river–distance strata. The figure provides a descriptive comparison of taxon-richness accumulation and sampling completeness across the aggregated strata.

Rarefaction/extrapolation supported the same richness ranking as the alpha-diversity point estimates: the 15 m strata, especially Łutownia 15 m, reached the highest taxon richness among the six river–distance strata (Figure 6; Table 2 and Table 3). Across all strata, the high Good’s coverage values (0.985–0.994) indicate that the aggregated samples captured most of the assemblage detectable at the present analytical resolution. Additional iNEXT outputs and rarefied-richness values are provided in the Supplementary Materials from [23].

### 3.3. Taxonomic Composition

**Figure 7:**
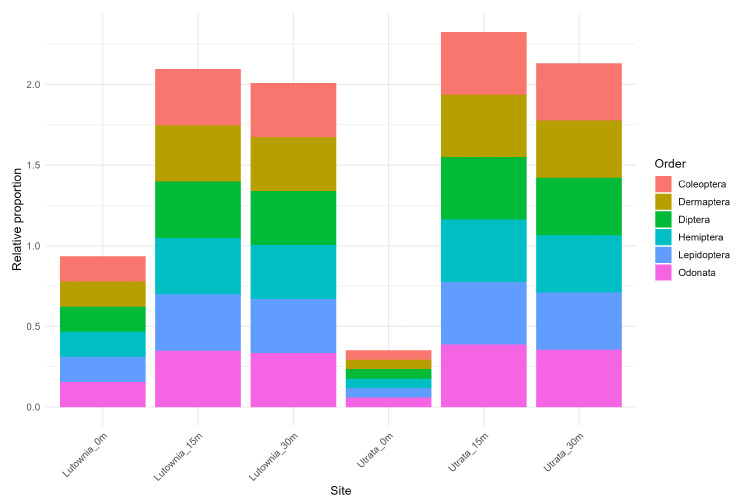
Taxonomic composition by order. Stacked proportions highlight shifts in the contribution of major insect orders among river × distance strata, showing that assemblage differences involved broad taxonomic reorganisation rather than only changes in total abundance.

Order-level proportions indicate that differences among river–distance strata involved changes in broad taxonomic structure as well as in total richness and abundance (Figure 7). Coleoptera, Diptera, Hymenoptera, Lepidoptera, Hemiptera and Odonata contributed unevenly among strata, consistent with the broader representation of aquatic, wet-riparian and dead-wood-associated taxa in the Łutownia assemblage and the more restricted taxonomic profile of Utrata.

### 3.4. Taxon-Level Abundance by Distance

**Figure 8:**
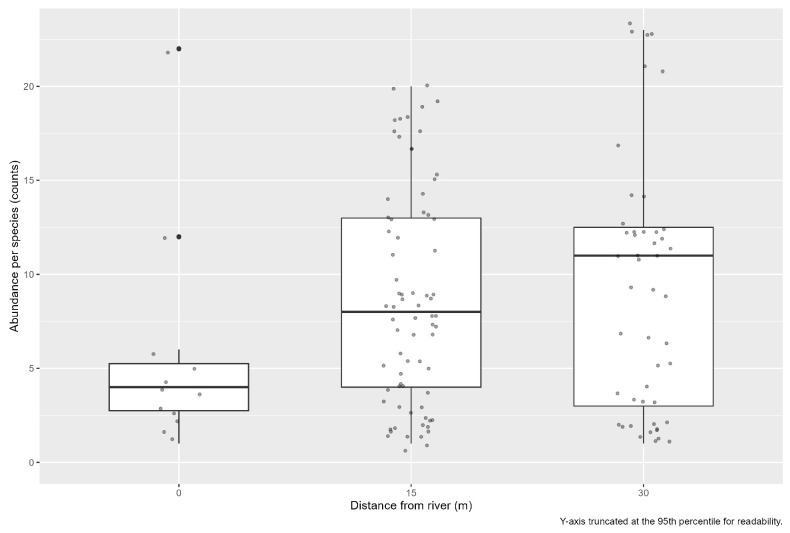
Taxon-level abundance by distance—Utrata. Boxplots of non-zero per-taxon counts at 0, 15 and 30 m. The y-axis is truncated at the river-specific 95th percentile; points indicate individual taxon values.

**Figure 9:**
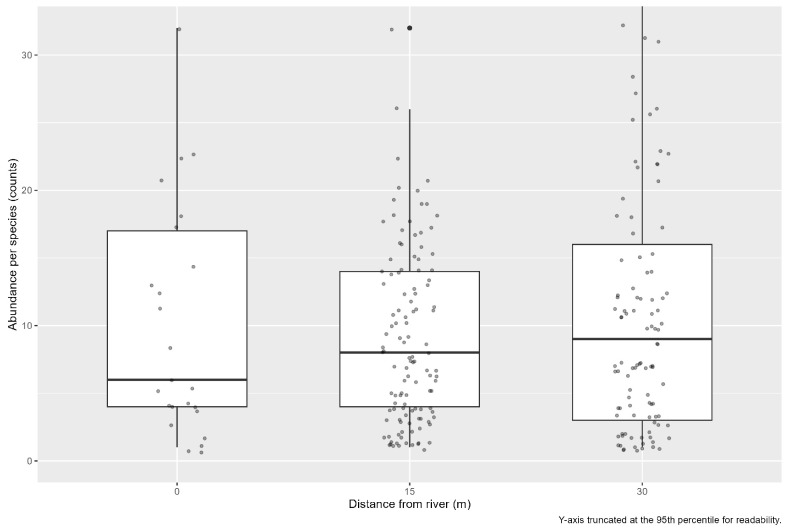
Taxon-level abundance by distance—Łutownia. Boxplots of non-zero per-taxon counts at 0, 15 and 30 m. The y-axis is truncated at the river-specific 95th percentile; points indicate individual taxon values.

Non-zero per-taxon abundance distributions also changed with distance from the channel (Figure 8 and Figure 9). In Utrata, typical non-zero taxon abundance increased from the river edge towards the outer zones, indicating that many taxa were represented by larger local counts away from the immediate channel margin. In Łutownia, central tendency also rose slightly with distance, but the most conspicuous feature was the broader upper tail at 30 m, where a few taxa reached particularly high counts. Thus, the outer Łutownia stratum was characterised by stronger dominance within the pooled assemblage, whereas Utrata showed a more gradual increase in typical non-zero counts away from the channel margin.

### 3.5. Beta Diversity

**Figure 10:**
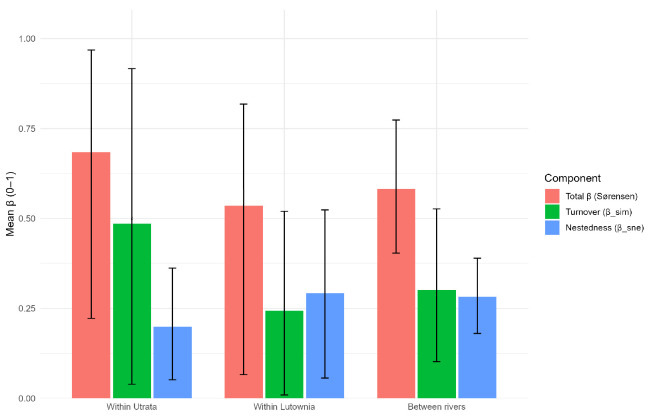
Beta diversity (Sørensen) and components. Mean Total β (Sørensen), turnover ($\beta_{\mathrm{sim}}$) and nestedness ($\beta_{\mathrm{sne}}$) for Within Utrata, Within Łutownia and Between rivers, with 95% percentile bootstrap intervals (2000 resamples).

The β-diversity decomposition likewise showed marked compositional differentiation among strata (Table 4; Figure 10). Within Utrata, mean Sørensen dissimilarity was highest and was driven mainly by turnover, pointing to substantial taxon replacement among distance zones. Within the Łutownia valley, mean dissimilarity was slightly lower and split more evenly between turnover and nestedness, whereas the between-river comparison was intermediate and also reflected contributions from both components. Pairwise values were nevertheless highly heterogeneous, ranging from very low dissimilarity between the 15 m and 30 m strata in Łutownia to very high dissimilarity between Łutownia 0 m and Utrata 30 m. Thus, the observed compositional pattern varied with both river identity and position along the short channel-distance gradient. The full set of pairwise values is provided in the Supplementary Materials from [23].

### 3.6. NMDS Ordination

**Figure 11:**
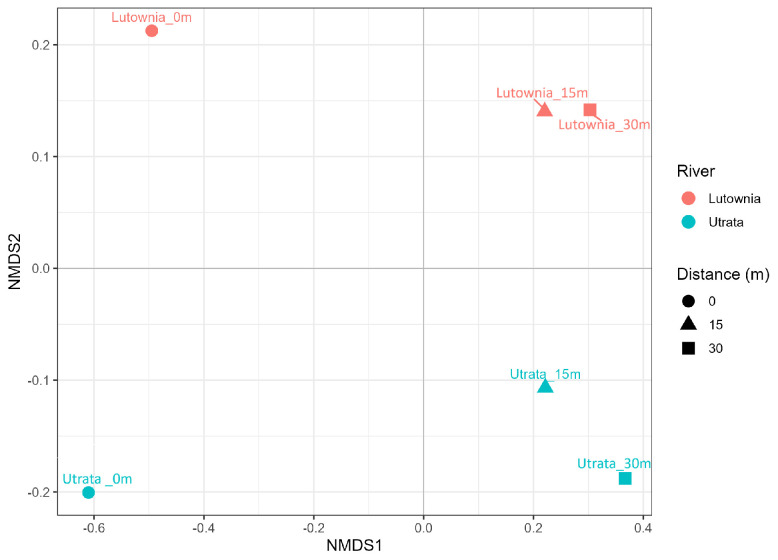
Exploratory NMDS ordination of the six river–distance strata based on square-root-transformed abundance data and Bray–Curtis dissimilarity. The two-dimensional solution had stress =0.

The exploratory NMDS ordination based on square-root-transformed abundances and Bray–Curtis dissimilarities yielded a two-dimensional solution with stress =0 (Figure 11). Thus, the rank-order dissimilarities among the six aggregated strata were represented exactly in two dimensions. Given the small number of analytical units and the aggregated nature of the matrix, the ordination is used here as a compact visual synthesis of among-stratum relationships. The corresponding site scores are provided in the Supplementary Materials from [23].

### 3.7. Family-Level Taxon Richness

**Figure 12:**
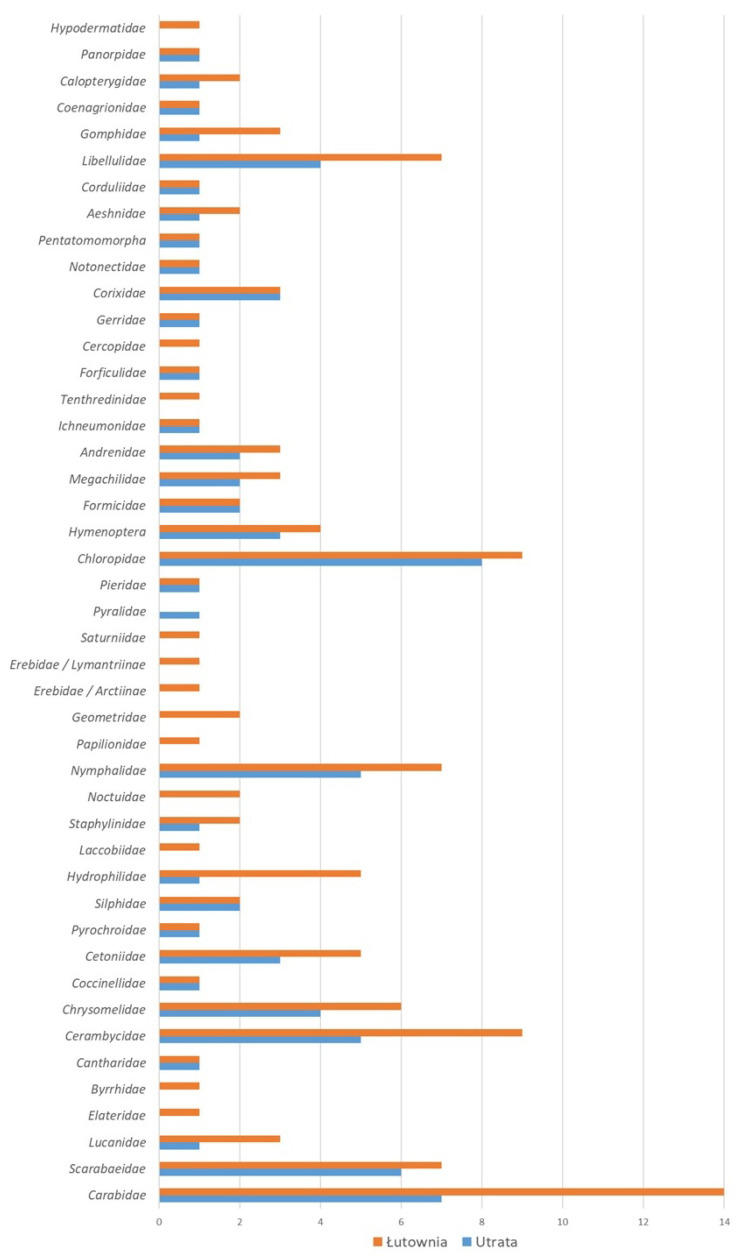
Number of insect taxa assigned to individual families recorded in the Utrata River valley and in the Łutownia River valley.

Family-level richness reinforced the between-valley contrast detected at the taxon level. The Łutownia valley had a broader family-level profile, with several beetle, odonate and lepidopteran families represented by more taxa than in Utrata (Figure 12). This pattern matches the presence of additional dead-wood-associated, aquatic and wet-riparian taxa in Łutownia and indicates that the higher richness in that valley was distributed across multiple families rather than being concentrated in a single taxonomic group.

## 4. Discussion

Alpha-diversity metrics and their bootstrap intervals identified pronounced spatial heterogeneity over short distances from the river edge, with a clear 15 m maximum in both valleys (Table 2 and Table 3; Figure 5). This intermediate-distance peak indicates that the zone slightly away from the immediate channel margin supported the richest and most even assemblages within the sampled riparian mosaic. Patterns at 30 m differed between rivers: Utrata retained intermediate diversity, whereas Łutownia showed high richness but lower evenness and inverse Simpson diversity, indicating stronger dominance by common taxa. The distance–zone pattern is therefore best interpreted as an assemblage-level association within the sampled habitat mosaic rather than as evidence for a single microhabitat driver. This reading is broadly consistent with work from natural temperate riparian forests showing high densities and diversity of tree-related microhabitats where living and dead trees create a fine-grained habitat mosaic [24]. As expected for effective numbers, inverse Simpson values (q=2) were consistently lower than Shannon values (q=1), confirming that dominance structure varied among strata. Together, these metrics provide a comparative picture of short-distance variation in assemblage structure around declining riparian alder stands.

Order-level proportional summaries and the family-level richness profile provide a complementary, coarser view of assemblage structure. Although they do not resolve species-level patterns, they help to interpret the alpha-diversity results as shifts in the contribution of higher taxonomic groups among river–distance strata.

Taxon-level abundance distributions based on non-zero counts also changed with distance from the channel, particularly in Utrata, where typical per-taxon abundance increased away from the river edge. In the Łutownia valley, the central tendency changed less, but the outer zone showed a much broader upper tail, indicating local dominance by a few taxa. Comparable riparian studies have reported shifts towards more opportunistic assemblages under disturbance [25]. In the present dataset, the broader upper tail at 30 m in Łutownia indicates that high abundance in the outer zone was concentrated in a subset of taxa, whereas Utrata showed a more gradual distance-related increase in typical non-zero counts. This contrast complements the alpha-diversity results by showing that distance-related patterns involved both richness and dominance structure.

The β-diversity summaries likewise indicated strong compositional differentiation among strata (Table 4; Figure 10). Within Utrata, mean dissimilarity was highest and was driven primarily by turnover, suggesting substantial taxon replacement across distances. Within the Łutownia valley, mean dissimilarity was slightly lower and was split between turnover and nestedness. Between-river dissimilarity was intermediate, with comparable contributions from both components. Wide bootstrap intervals and highly heterogeneous pairwise values (e.g., very low dissimilarity between Łutownia 15 m and 30 m versus very high dissimilarity between Łutownia 0 m and Utrata 30 m) showed that assemblage differences were strongly context dependent across specific stratum pairings. The exploratory NMDS ordination provided a complementary abundance-based view of these relationships (Figure 11). Its stress-free two-dimensional solution indicates that the Bray–Curtis relationships among the six aggregated strata can be displayed without distortion; however, because the ordination was based on a very small number of pooled units, it should be read as a compact visual synthesis of the comparative relationships among strata. At the same time, the consistently high Good’s coverage values and the rarefaction/extrapolation curves suggest that the main between-stratum differences are unlikely to arise solely from incomplete sampling at the analytical resolution used here.

Within-season differences were visible mainly in the identity of prominent taxa rather than in strong fluctuations of total captures. The near-natural Łutownia valley maintained higher pooled point-level totals throughout the sampling period, whereas Utrata remained consistently poorer and more strongly represented by widespread generalists. Thus, the between-valley contrast was more pronounced and more consistent than the short-term shifts among campaigns. Because the study covered summer and early autumn and Łutownia points were visited in alternating monthly subsets, temporal patterns in that valley are interpreted at the campaign level.

At the landscape scale, the contrast between the near-natural Łutownia valley within the Białowieża Forest and the urbanised Utrata valley accords with broader European evidence that riparian and alder forests retain higher ecological value where structural continuity, riparian forest cover and dead wood are preserved [24,26]. In declining alder stands in Poland, previous work has documented beetle assemblages associated with declining and recently dead *Alnus glutinosa* and has highlighted the importance of such trees for saproxylic fauna [10,27]. These earlier alder studies focused mainly on insects directly associated with affected trees, whereas the present sampling additionally captured the surrounding ground and vegetation layers of the riparian habitat mosaic. In southern European riparian alder forests, disturbance has likewise been associated with a shift towards more mobile and opportunistic carabid assemblages [25], while in the Po plain, frequent coppicing simplified black-alder stand structure and worsened habitat conservation status [26]. Similarly, saproxylic beetle assemblages in northern Italian floodplain forests were poorer and more generalist where deadwood was regularly removed, whereas mature, better-preserved stands supported richer communities [28]. The richer Łutownia assemblage therefore matches the broader pattern expected for less transformed riparian forests, whereas the Utrata assemblage is more consistent with a fragmented valley exposed to strong anthropogenic disturbance.

These findings have implications for riparian management and saproxylic conservation when interpreted together with the broader literature. The present descriptive comparison is consistent with retaining riparian forest belts as structurally diverse habitat zones rather than reducing them to narrow bank strips and with retaining a range of dead wood forms—including standing dead alder, fallen trunks, large branches and partly decayed material—where public-safety and flood-conveyance constraints allow. This interpretation is consistent with European evidence linking saproxylic richness to dead-wood amount, continuity and variety [28,29,30]. In near-natural valleys such as Łutownia, this mainly means avoiding unnecessary removal of naturally generated dead wood. In transformed valleys such as Utrata, it suggests restoration measures that improve riparian continuity, reduce hydromorphological simplification and retain selected dead-wood structures instead of routine blanket sanitation felling.

Several aspects of the study design define the scale of inference. The study compared two river systems during one June–September growing season, with balanced seasonal point visits but different numbers of unique sampling points between rivers. The design captured insects active on the ground, in low vegetation and on accessible dead-wood surfaces, whereas canopy and upper-trunk specialists were less well represented. Tree condition and individual microhabitat availability were not quantified, so the analyses describe assemblage patterns associated with declining alder stands and their composite riparian habitat mosaic rather than estimating specific dead-wood thresholds or tree-level effects. Retained non-protected specimens are housed in the reference collection of Konrad Wilamowski at the Institute of Forest Sciences, Białystok University of Technology, rather than in a formal institutional collection, which limits long-term independent verification. These constraints do not alter the main comparative result: the near-natural Łutownia valley supported a richer and taxonomically broader assemblage than the urbanised Utrata valley, with strong short-distance variation in diversity and composition.

Declining alder stands in riparian valleys can concentrate habitat heterogeneity through fresh dead wood, loosening bark, fungal fruiting bodies, newly exposed saproxylic substrates and partial canopy opening. In the present comparison, the richer Łutownia assemblage, the intermediate-distance diversity maximum and the compositional differentiation among strata indicate that such riparian alder mosaics can support spatially structured insect assemblages. The broader literature further indicates that durable conservation value depends on continuity of diverse dead wood through successive stages of decomposition [29,30,31]. The highest conservation value is therefore likely to occur where early alder decline is embedded within a riparian forest that retains both standing and fallen wood across multiple decay stages. Taken together with published evidence, the fine-scale distance patterns and between-valley contrasts support the importance of maintaining structurally complex, continuous riparian habitats and mitigating key urban pressures to conserve river-valley insect assemblages.

## 5. Conclusions

Insect assemblages associated with declining riparian black alder stands differed clearly between the near-natural Łutownia valley and the urbanised Utrata valley. The Łutownia valley supported a richer and taxonomically broader assemblage, with a stronger representation of forest-associated, saproxylic and aquatic taxa, whereas Utrata was dominated more strongly by widespread disturbance-tolerant taxa. Diversity peaked at 15 m from the channel margin in both valleys, and the between-valley contrast was stronger than the within-season variation recorded during the study. Because tree-level attributes and individual microhabitat categories were not quantified separately, microhabitat-related conclusions are interpreted as sampling-point-scale associations within the riparian habitat mosaic. The main contribution of the study is therefore to show that declining alder stands in structurally complex riparian corridors support distinctive insect assemblages shaped by valley context and short-distance position relative to the river channel.

## Figures and Tables

**Table 1:** Approximate geographic coordinates and brief descriptions of the sampling points located along the Utrata and Łutownia rivers. Coordinates are provided in the WGS 84 system and rounded to four decimal places (approximately 10 m precision) to represent reference locations for the sampled areas. The table summarises the spatial layout of the sampled areas in two contrasting river valleys: the urbanised Utrata valley and the near-natural Łutownia valley.

River	Point	Approximate Latitude (N)	Approximate Longitude (E)	Description of Location
Utrata	PU1	52.0974	20.8431	Midstream section; declining bank alders; close to buildings
Utrata	PU2	52.0928	20.8405	Semi-natural section adjacent to urban areas
Utrata	PU3	52.0968	20.8504	Valley fragment with well-developed riparian vegetation
Łutownia	PŁ1	52.7336	23.7897	Upper valley section near the spring area
Łutownia	PŁ2	52.7282	23.7476	Dense stand with abundant dead wood
Łutownia	PŁ3	52.7357	23.7639	Transition zone from alder forest to low bog
Łutownia	PŁ4	52.7440	23.7500	Fragment with sparse trees and shrubby undergrowth
Łutownia	PŁ5	52.7460	23.7164	Lower valley section near wet meadows
Łutownia	PŁ6	52.7615	23.6954	Section with numerous declining alders

**Table 2:** Alpha-diversity point estimates by river–distance stratum.

River	Distance (m)	*N*	$S_{\mathrm{obs}}$	$H^{\prime }$	exp($H^{\prime }$)	1 − D	1/D	$J^{\prime }$	Good’s Coverage
Utrata	0	322	12	2.122	8.35	0.835	6.05	0.854	0.985
Utrata	15	363	75	3.919	50.35	0.967	30.42	0.908	0.993
Utrata	30	305	51	3.339	28.19	0.939	16.42	0.849	0.994
Łutownia	0	594	25	2.784	16.19	0.918	12.13	0.865	0.990
Łutownia	15	631	120	4.231	68.80	0.967	30.22	0.884	0.991
Łutownia	30	561	107	3.513	33.54	0.893	9.32	0.752	0.994

**Table 3:** Selected alpha-diversity metrics with 95% percentile bootstrap intervals by river–distance stratum. Values are reported as estimate (95% percentile bootstrap interval). For observed richness, the percentile interval describes the bootstrap resampling distribution under fixed total abundance and may therefore lie below the original observed value.

River	Distance (m)	$S_{\mathrm{obs}}$	exp($H^{\prime }$)	1/D	$J^{\prime }$	Good’s Coverage
Utrata	0	12 (10–12)	8.35 (6.06–9.30)	6.05 (4.11–7.68)	0.854 (0.761–0.911)	0.985 (0.941–1.000)
Utrata	15	75 (68–74)	50.35 (44.51–50.97)	30.42 (24.62–34.64)	0.908 (0.890–0.921)	0.993 (0.988–0.997)
Utrata	30	51 (45–51)	28.19 (24.86–29.39)	16.42 (13.89–18.56)	0.849 (0.830–0.872)	0.994 (0.988–0.998)
Łutownia	0	25 (22–25)	16.19 (14.00–17.08)	12.13 (9.98–13.59)	0.865 (0.836–0.897)	0.990 (0.983–1.000)
Łutownia	15	120 (110–118)	68.80 (61.37–69.90)	30.22 (25.18–34.89)	0.884 (0.869–0.896)	0.991 (0.989–0.997)
Łutownia	30	107 (97–105)	33.54 (30.06–35.27)	9.32 (8.31–10.44)	0.752 (0.737–0.771)	0.994 (0.993–0.998)

**Table 4:** Summary of Sørensen beta diversity and its components by comparison category. Values are reported as mean (95% percentile bootstrap interval).

Comparison Category	Component	*n* Pairs	Mean	95% Bootstrap Interval
Between rivers	Total $\beta_{\mathrm{sor}}$	9	0.583	0.397–0.769
Between rivers	Turnover $\beta_{\mathrm{sim}}$	9	0.301	0.111–0.526
Between rivers	Nestedness $\beta_{\mathrm{sne}}$	9	0.282	0.190–0.384
Within Łutownia	Total $\beta_{\mathrm{sor}}$	3	0.536	0.066–0.818
Within Łutownia	Turnover $\beta_{\mathrm{sim}}$	3	0.243	0.009–0.520
Within Łutownia	Nestedness $\beta_{\mathrm{sne}}$	3	0.293	0.057–0.524
Within Utrata	Total $\beta_{\mathrm{sor}}$	3	0.684	0.222–0.968
Within Utrata	Turnover $\beta_{\mathrm{sim}}$	3	0.485	0.039–0.917
Within Utrata	Nestedness $\beta_{\mathrm{sne}}$	3	0.199	0.052–0.362

## Data Availability

The original contributions presented in this study are included in the article. The cleaned taxon-by-stratum matrix, supplementary statistical outputs underlying the community-level analyses, and reproducible R code for the statistical workflow are publicly available in Mendeley Data [23]. Retained non-protected specimens are housed in the reference collection of Konrad Wilamowski at the Institute of Forest Sciences, Białystok University of Technology. Further inquiries can be directed to the corresponding author.

## Appendix A

**Table A1:** Presence/absence of selected insect taxa recorded in the Utrata and Łutownia river valleys. Entries are reported at the species level where possible and otherwise at the lowest reliable taxonomic rank used in the dataset. The symbol “+” indicates that the taxon was recorded at a given sampling location, whereas “–” indicates that it was not recorded there.

Order	Family	Taxon	Utrata	Łutownia
Coleoptera	Carabidae	*Agonum dorsale*	–	+
		*Amara aenea*	–	+
		*Anchomenus dorsalis*	–	+
		*Bembidion properans*	+	+
		*Calosoma inquisitor*	+	+
		*Carabus granulatus*	+	+
		*Harpalus affinis*	–	+
		*Leistus ferrugineus*	–	+
		*Nebria brevicollis*	+	+
		*Notiophilus biguttatus*	+	+
		*Poecilus cupreus*	–	+
		*Pterostichus melanarius*	+	+
		*Pterostichus niger*	+	+
		*Scarites subterraneus*	–	+
Coleoptera	Scarabaeidae	*Anisoplia austriaca*	–	+
		*Aphodius fossor*	+	+
		*Melolontha melolontha*	+	+
		*Melolontha hippocastani*	+	+
		*Phyllopertha horticola*	+	+
Coleoptera	Geotrupidae	*Anoplotrupes stercorosus*	+	+
		*Geotrupes stercorarius*	+	+
Coleoptera	Lucanidae	*Dorcus parallelipipedus*	+	+
		*Platycerus caraboides*	–	+
		*Sinodendron cylindricum*	–	+
Coleoptera	Elateridae	*Elater ferrugineus*	–	+
Coleoptera	Byrrhidae	*Byrrhus pilula*	–	+
Coleoptera	Cantharidae	*Cantharis rustica*	+	+
Coleoptera	Cerambycidae	*Corymbia rubra*	+	+
		*Macroleptura thoracica*	+	+
		*Lamia textor*	–	+
		*Prionus coriarius*	–	+
		*Rhagium inquisitor*	–	+
		*Rhagium mordax*	+	+
		*Saperda scalaris*	–	+
		*Rutpela maculata*	+	+
Coleoptera	Chrysomelidae	*Agelastica alni*	+	+
		*Donacia semicuprea*	–	+
		*Galeruca pomonae*	–	+
		*Galeruca tanaceti*	+	+
		*Plagiosterna aenea*	+	+
Coleoptera	Curculionidae	*Sitona lineatus*	+	+
Coleoptera	Coccinellidae	*Coccinella septempunctata*	+	+
		*Harmonia axyridis*	+	+
		*Propylea quatuordecimpunctata*	–	+
Coleoptera	Scarabaeidae (Cetoniinae)	*Cetonia aurata*	+	+
		*Oxythyrea funesta*	+	+
		*Protaetia (Cetonischema) speciosissima*	+	+
		*Trichius gallicus*	–	+
		*Tropinota (Epicometis) hirta*	–	+
Coleoptera	Pyrochroidae	*Pyrochroa coccinea*	+	+
Coleoptera	Silphidae	*Oiceoptoma thoracicum*	+	+
		*Silpha obscura*	+	+
Coleoptera	Dytiscidae	*Dytiscus marginalis*	+	+
Coleoptera	Hydrophilidae	*Hydrophilus aterrimus*	–	+
		*Hydrophilus chrysomelinus*	–	+
		*Hydrochara caraboides*	–	+
		*Hydrophilus piceus*	+	+
		*Laccobius minutus*	–	+
Coleoptera	Staphylinidae	*Staphylinus erythropterus*	+	+
		*Staphylinus dimidiaticornis*	–	+
Lepidoptera	Noctuidae	*Acronicta cuspis*	–	+
		*Acronicta psi*	–	+
Lepidoptera	Nymphalidae	*Aglais io*	+	+
		*Aglais urticae*	+	+
		*Nymphalis antiopa*	–	+
		*Vanessa atalanta*	+	+
		*Vanessa cardui*	+	+
Lepidoptera	Pieridae	*Gonepteryx rhamni*	+	+
		*Pieris brassicae*	+	+
Lepidoptera	Lycaenidae	*Polyommatus icarus*	–	+
Lepidoptera	Papilionidae	*Papilio machaon*	–	+
Lepidoptera	Geometridae	*Hydriomena furcata*	–	+
		*Lomaspilis marginata*	–	+
Lepidoptera	Erebidae (Arctiinae)	*Arctia caja*	–	+
Lepidoptera	Erebidae (Lymantriinae)	*Euproctis chrysorrhoea*	–	+
Lepidoptera	Saturniidae	*Saturnia pyri*	–	+
Lepidoptera	Pyralidae	*Plodia interpunctella*	+	–
Diptera	Tabanidae	*Chrysops caecutiens*	+	+
		*Haematopota pluvialis*	+	+
		*Tabanus bovinus*	–	+
Diptera	Stratiomyidae	Stratiomyidae sp.	+	+
Diptera	Simuliidae	Simuliidae sp.	+	+
Diptera	Tipulidae	*Tipula* sp.	+	+
Diptera	Limoniidae	Limoniidae sp.	+	+
Diptera	Dolichopodidae	Dolichopodidae sp.	+	+
Diptera	Syrphidae	*Eristalis tenax*	+	+
Hymenoptera	Apidae	*Apis mellifera*	+	+
		*Bombus hypnorum*	+	+
		*Bombus lapidarius*	–	+
		*Bombus terrestris*	+	+
Hymenoptera	Formicidae	*Formica rufa*	+	+
		*Lasius niger*	+	+
Hymenoptera	Megachilidae	*Osmia bicornis*	+	+
		*Chelostoma florisomne*	–	+
		*Megachile ligniseca*	+	+
Hymenoptera	Andrenidae	*Andrena vaga*	+	+
		*Andrena fulva*	–	+
		*Andrena flavipes*	+	–
Hymenoptera	Ichneumonidae	Ichneumonidae sp.	+	+
Hymenoptera	Tenthredinidae	*Tenthredo* sp.	–	–
Dermaptera	Forficulidae	*Forficula auricularia*	+	+
Hemiptera	Cercopidae	*Cercopis vulnerata*	–	+
Hemiptera	Gerridae	*Gerris lacustris*	+	+
		*Velia caprai*	–	+
Hemiptera	Corixidae	*Arctocorisa germari*	+	+
		*Corixa punctata*	+	+
		*Sigara striata*	+	+
Hemiptera	Notonectidae	*Notonecta glauca*	+	+
Hemiptera	Pyrrhocoridae	*Pyrrhocoris apterus*	+	+
Odonata	Aeshnidae	*Aeshna cyanea*	+	+
		*Aeshna juncea*	–	+
Odonata	Corduliidae	*Cordulia aenea*	+	+
Odonata	Libellulidae	*Crocothemis erythraea*	–	+
		*Libellula depressa*	+	+
		*Orthetrum cancellatum*	+	+
		*Sympetrum fonscolombii*	–	+
		*Sympetrum sanguineum*	–	+
		*Sympetrum striolatum*	+	+
		*Sympetrum vulgatum*	+	+
Odonata	Gomphidae	*Gomphus flavipes*	–	+
		*Gomphus vulgatissimus*	+	+
		*Ophiogomphus cecilia*	–	+
Odonata	Coenagrionidae	*Enallagma cyathigerum*	+	+
Odonata	Calopterygidae	*Calopteryx virgo*	+	+
		*Calopteryx splendens*	–	+
Mecoptera	Panorpidae	*Panorpa communis*	+	+
Diptera	Oestridae	*Hypoderma bovis*	–	+

**Table A2:** Raw point-level counts and dominant taxa recorded in the Utrata River valley (sampling points PU1–PU3) in 2024. The table is stratified by distance zone from the channel margin and summarises individual point–distance combinations pooled across sampling methods.

Month	Point	Distance from Channel Margin (m)	Number of Individuals	Dominant Taxa
June	PU1	0	28	*Lasius niger*, *Formica rufa*
	PU1	15	32	*Coccinella septempunctata*, *Tipula* sp.
	PU1	30	25	*Lasius niger*, *Chrysoperla carnea*
	PU2	0	26	*Pterostichus melanarius*, *Formica rufa*
	PU2	15	30	*Lasius niger*, *Carabus granulatus*
	PU2	30	24	*Tipula* sp., *Coccinella septempunctata*
	PU3	0	29	*Lasius niger*, *Hydrophilus piceus*
	PU3	15	31	*Formica rufa*, *Chrysoperla carnea*
	PU3	30	27	*Tipula* sp., *Carabus granulatus*
July	PU1	0	30	*Staphylinus erythropterus*, *Lasius niger*
	PU1	15	34	*Formica rufa*, *Carabus granulatus*
	PU1	30	26	*Tipula* sp., *Coccinella septempunctata*
	PU2	0	28	*Pterostichus melanarius*, *Chrysoperla carnea*
	PU2	15	30	*Formica rufa*, *Lasius niger*
	PU2	30	27	*Tipula* sp., *Carabus granulatus*
	PU3	0	30	*Lasius niger*, *Hydrophilus piceus*
	PU3	15	33	*Formica rufa*, *Chrysoperla carnea*
	PU3	30	29	*Carabus granulatus*, *Tipula* sp.
August	PU1	0	26	*Lasius niger*, *Aeshna cyanea*
	PU1	15	29	*Carabus granulatus*, *Chrysoperla carnea*
	PU1	30	25	*Formica rufa*, *Tipula* sp.
	PU2	0	23	*Pterostichus melanarius*, *Formica rufa*
	PU2	15	28	*Lasius niger*, *Carabus granulatus*
	PU2	30	25	*Tipula* sp., *Chrysoperla carnea*
	PU3	0	27	*Hydrophilus piceus*, *Formica rufa*
	PU3	15	29	*Chrysoperla carnea*, *Aeshna cyanea*
	PU3	30	26	*Formica rufa*, *Tipula* sp.
September	PU1	0	24	*Coccinella septempunctata*, *Hydrophilus piceus*
	PU1	15	28	*Lasius niger*, *Formica rufa*
	PU1	30	22	*Chrysoperla carnea*, *Aeshna cyanea*
	PU2	0	25	*Pterostichus melanarius*, *Carabus granulatus*
	PU2	15	29	*Formica rufa*, *Tipula* sp.
	PU2	30	24	*Lasius niger*, *Chrysoperla carnea*
	PU3	0	26	*Hydrophilus piceus*, *Aeshna cyanea*
	PU3	15	30	*Chrysoperla carnea*, *Formica rufa*
	PU3	30	25	*Tipula* sp., *Carabus granulatus*

**Table A3:** Raw point-level counts and dominant taxa recorded in the Łutownia River valley (sampling points PŁ1–PŁ6) in 2024. The table is stratified by distance zone from the channel margin and summarises individual point–distance combinations pooled across sampling methods.

Month	Point	Distance from Channel Margin (m)	Number of Individuals	Dominant Taxa
June	PŁ1	0	45	*Carabus granulatus*, *Chrysoperla carnea*
	PŁ1	15	51	*Formica rufa*, *Aeshna cyanea*
	PŁ1	30	43	*Donacia clavipes*, *Hydrophilus piceus*
	PŁ2	0	50	*Tipula* sp., *Carabus granulatus*
	PŁ2	15	47	*Aeshna juncea*, *Lasius niger*
	PŁ2	30	44	*Formica rufa*, *Agelastica alni*
	PŁ3	0	49	*Cordulia aenea*, *Anoplotrupes stercorosus*
	PŁ3	15	53	*Gomphus vulgatissimus*, *Lucanus cervus*
	PŁ3	30	46	*Limnius volckmari*, *Chrysoperla carnea*
July	PŁ4	0	52	*Sympetrum sanguineum*, *Dorcus parallelipipedus*
	PŁ4	15	55	*Formica rufa*, *Calosoma inquisitor*
	PŁ4	30	48	*Pterostichus melanarius*, *Oedemera nobilis*
	PŁ5	0	49	*Lasius niger*, *Hydrophilus piceus*
	PŁ5	15	50	*Aeshna cyanea*, *Carabus granulatus*
	PŁ5	30	47	*Donacia simplex*, *Staphylinus erythropterus*
	PŁ6	0	53	*Aeshna juncea*, *Sympetrum vulgatum*
	PŁ6	15	55	*Chrysoperla carnea*, *Hydrophilus piceus*
	PŁ6	30	50	*Eristalis tenax*, *Polyommatus icarus*
August	PŁ1	0	48	*Pieris brassicae*, *Hydrophilus piceus*
	PŁ1	15	52	*Lasius niger*, *Aeshna cyanea*
	PŁ1	30	46	*Formica rufa*, *Donacia simplex*
	PŁ2	0	50	*Melolontha melolontha*, *Carabus granulatus*
	PŁ2	15	54	*Lucanus cervus*, *Anoplotrupes stercorosus*
	PŁ2	30	48	*Cordulia aenea*, *Hydrophilus piceus*
	PŁ3	0	53	*Aeshna juncea*, *Limnius volckmari*
	PŁ3	15	57	*Tipula* sp., *Gomphus flavipes*
	PŁ3	30	49	*Formica rufa*, *Chrysoperla carnea*
September	PŁ4	0	46	*Vanessa atalanta*, *Aeshna cyanea*
	PŁ4	15	50	*Carabus granulatus*, *Hydrophilus piceus*
	PŁ4	30	45	*Limnius volckmari*, *Anoplotrupes stercorosus*
	PŁ5	0	49	*Dorcus parallelipipedus*, *Formica rufa*
	PŁ5	15	53	*Chrysoperla carnea*, *Donacia simplex*
	PŁ5	30	47	*Carabus granulatus*, *Tipula* sp.
	PŁ6	0	50	*Gomphus vulgatissimus*, *Hydrophilus piceus*
	PŁ6	15	54	*Sympetrum striolatum*, *Lucanus cervus*
	PŁ6	30	48	*Chrysoperla carnea*, *Formica rufa*
